# Developmental Differences of Structural Connectivity and Effective Connectivity in Semantic Judgments of Chinese Characters

**DOI:** 10.3389/fnhum.2020.00233

**Published:** 2020-06-30

**Authors:** Li-Ying Fan, Yu-Chun Lo, Yung-Chin Hsu, Yu-Jen Chen, Wen-Yih Isaac Tseng, Tai-Li Chou

**Affiliations:** ^1^Department of Education, National Taipei University of Education, Taipei, Taiwan; ^2^Department of Psychology, National Taiwan University, Taipei, Taiwan; ^3^Department of Thanatology and Health Counseling, National Taipei University of Nursing and Health Sciences, Taipei, Taiwan; ^4^Institute of Medical Device and Imaging, National Taiwan University College of Medicine, Taipei, Taiwan; ^5^The Ph.D. Program for Neural Regenerative Medicine, College of Medical Science and Technology, Taipei Medical University, Taipei, Taiwan; ^6^Research Center of Brain and Consciousness, Taipei Medical University, Taipei, Taiwan; ^7^Graduate Institute of Brain and Mind Sciences, National Taiwan University, Taipei, Taiwan; ^8^Molecular Imaging Center, National Taiwan University, Taipei, Taiwan; ^9^Center for Advanced Study in the Behavioral Sciences, Stanford University, Stanford, CA, United States

**Keywords:** meaning, semantics, structural connectivity, effective connectivity, development

## Abstract

Previous studies have investigated the developmental differences of semantic processing regarding brain activation between adults and children. However, little is known about whether the patterns of structural connectivity and effective connectivity differ between adults and children during semantic processing. Functional magnetic resonance imaging (fMRI), diffusion spectrum imaging (DSI), and dynamic causal modeling (DCM) were used to study the developmental differences of brain activation, structural connectivity, and effective connectivity during semantic judgments. Twenty-six children (8- to 12-year-olds) and 26 adults were asked to indicate if character pairs were related in meaning. Compared to children, adults showed greater activation in the left ventral inferior frontal gyrus (IFG) and left middle temporal gyrus (MTG). Also, adults had significantly greater structural connectivity in the left ventral pathway (inferior frontal occipital fasciculus, IFOF) than children. Moreover, adults showed significantly stronger bottom-up effects from left fusiform gyrus (FG) to ventral IFG than children in the related condition. In conclusion, our findings suggest that age-related increases in brain activation (ventral IFG and MTG), IFOF, and effective connectivity (from FG to ventral IFG) might be associated with the bottom-up influence of orthographic representations on retrieving semantic representations for processing Chinese characters.

## Introduction

Previous functional and structural studies have proposed that a dual-stream model for the language network includes the ventral and dorsal pathways that connect the functionally relevant frontal and temporal regions (Saur et al., 2008). The dorsal pathway connects the opercular part of the IFG (dorsal IFG) and the superior temporal gyrus (STG) *via* the arcuate fasciculus (AF) and the superior longitudinal fasciculus (SLF), while the ventral pathway connects the orbital part of IFG (ventral IFG), the middle temporal gyrus (MTG), and the fusiform gyrus (FG) *via* the inferior frontal-occipital fasciculus (IFOF) and the inferior longitudinal fasciculus (ILF; Saur et al., 2008). Relevance to the structural division, previous studies have also suggested functional differences for the sub-parts of the left inferior frontal gyrus (IFG). The dorsal region of the left IFG is found to process phonological information, while the ventral region of the left IFG is associated with semantic processing (Poldrack et al., 1999; Liu et al., 2009).

Regarding developmental differences (ranging from 8 to 20 years) in brain activation, two major brain regions have been suggested to be associated with semantic processing as a network. These two brain regions include: the left ventral IFG and left MTG (Lau et al., 2008). The ventral IFG is associated with controlling semantic retrieval (Lau et al., 2008), while the left MTG is considered to be related to the storage of lexical representations (Hickok and Poeppel, 2007; Martin, 2007). Using a semantic association task, similar activations in the ventral IFG and the MTG have been observed in Chinese adults (Dong et al., 2005; Booth et al., 2006; Chou et al., 2009b) and children (Chou et al., 2009a). A child study has shown that children have similar activation in the bilateral inferior frontal gyri and left MTG as reported by previous studies of adults for printed words (Chou et al., 2006).

To investigate developmental differences in structural connectivity, diffusion tensor imaging (DTI) has been used to obtain information about neural fiber orientations. Previous DTI studies have demonstrated age-related fractional anisotropy (FA) changes (ranging from 5.6 to 29.2 years) in the genu and splenium of the corpus callosum, the inferior and SLF, the inferior and superior fronto-occipital fasciculus, cingulum, uncinate fasciculus, and corticospinal tract (Lebel et al., 2008). The age-related increase in the volume of white matter and FA has been interpreted as evidence of reflecting continued axonal myelination during childhood and adolescence (Paus et al., 2008; Schmithorst and Yuan, 2010). Also, a previous developmental study has shown that children have lower GFA in dorsal and ventral pathways than adults (Brauer et al., 2013). However, DTI may provide false orientations for crossing fibers because the diffusion model of DTI assumes Gaussian distribution of water molecular diffusion within each voxel. Due to the limitation of DTI, DTI tractography may not provide appropriate fiber pathways, especially in the frontal regions (Wedeen et al., 2005). To resolve diffusion profiles in different directions, diffusion spectrum imaging (DSI) acquires hundreds of diffusion-weighted images corresponding to evenly-spaced grid points in 3-D q-space (Wedeen et al., 2005). Since DSI is more sensitive to crossing fibers; it is used in the present study to detect the developmental differences in structural connectivity between adults and children.

To investigate the developmental differences in effective connectivity, dynamic causal modeling (DCM) was used. Previous studies have suggested that effective connectivity measures such as DCM (Friston et al., 2003; Stephan et al., 2010) can estimate inter-regional relationships in a neural network, for example, the direction of the causal influence and its change due to experimental interventions. Our previous work on effective connectivity has provided evidence to support that the left IFG is functionally connected with the left MTG during semantic judgments (Fan et al., 2010; Fan and Chou, 2012). Also, previous studies have suggested that the linkage of different modulatory effects between the top-down controlled retrieval and the bottom-up automatic processing interacts to mediate the access of lexical-semantic knowledge in the MTG (Rossell et al., 2001; Cohen et al., 2004). However, to the best of our knowledge, no study has investigated the developmental differences of semantic processing using effective connectivity.

Previous studies have combined data from DTI and functional magnetic resonance imaging (fMRI) to investigate brain injuries and the organization of brain functions. A combination of techniques can provide additional information about brain organization which might provide different conclusions from an approach using single imaging modality (Werring et al., 1998, 1999; Wieshmann et al., 2001; Olesen et al., 2003). To date, no study has investigated developmental differences in brain activation, structural connectivity, and effective connectivity to understand the underlying mechanisms of semantic processing between adults and children in one study. Therefore, in the present study, we used task fMRI to examine the brain activation of semantic processing, DSI to assess the microstructural integrity, and DCM to examine the directional influences among brain regions between two groups. By combining multiple modalities, our goal was to provide a complete insight into the function and structure of the developmental differences between adults and children during semantic processing in Chinese. Besides, we examined the correlation between various pertinent factors and age in months across all participants. We hypothesized that functional activation, microstructural integrity, and effective connectivity in adults might be greater than those in children and that the degree of maturation could be revealed by examining the correlation between age and behavioral performance, structural connectivity, and effective connectivity.

## Materials and Methods

### Participants

Twenty-six adults [mean age = 25.9 years, standard deviation (SD) = 4.8 years, 14 males] and 26 children (mean age = 9.8 years, SD = 1.5 years, age range from 8- to 12-year-olds, 14 males) participated in the MRI experiment. All participants were given an interview to ensure that they met the following inclusion criteria: (1) native Mandarin-Chinese speakers; (2) right-handedness; (3) free of neurological disease or psychiatric disorders; (4) no history of language or reading disabilities; (5) not taking medication affecting the central nervous system; and (6) no learning difficulty. Participants who had a clinical diagnosis of any other psychiatric disorders were excluded from the present study. After receiving detailed information on the experimental purpose and administration, informed consent was obtained. The informed consent procedures were approved by the Institutional Review Board of National Taiwan University Hospital. Both fMRI and DSI data were collected to examine developmental differences in brain activation, effective connectivity, and structural connectivity, respectively. The present study was approved by and following the guidelines of the local ethics committee (Research Ethics Committee at National Taiwan University Hospital).

### Functional MRI Acquisition and Analysis

#### Stimuli and Procedures

Character pairs were divided into the semantically related and unrelated conditions (Chou et al., 2009b). Forty-eight character pairs were semantically related according to their free association values (mean = 0.14, SD = 0.13, ranging from 0.73 to 0.01; Hue et al., 2005). Twenty-four character pairs were semantically unrelated with zero association values. Several lexical variables were controlled across related and unrelated conditions. First, all characters were monosyllabic. Second, the first and second characters did not share radicals. Third, the first and second characters together did not form a word (Wu and Liu, 1987; Sinica, 1998). Fourth, characters were matched for visual complexity (in terms of strokes per character) across conditions. Fifth, characters were matched for frequency across conditions (Wu and Liu, 1987). Sixth, the number of nouns (48–50%), verbs (23%), and adjectives (21–27%), based on their most frequent usage in Academia Sinica balanced corpus (Sinica, 1998), was matched across conditions. The correlation of character frequency or the measure of semantic relation (Chou et al., 2009a; Lee et al., 2009) with association strength was not significant indicating that association effects should not be due to frequency or semantic relation differences (*r* = −0.02, *p* > 0.05 and *r* = −0.05, *p* > 0.05, respectively).

In the meaning judgment task, two visual Chinese characters were presented sequentially (Chou et al., 2009a,b). The participant had to determine whether the character pairs were related in meaning. Trials lasted 4,500 ms and consisted of a solid square (500 ms), followed by the first character (800 ms), a 200 ms blank interval, and the second character (3,000 ms). The participants responded during the presentation of the second character. They were instructed to quickly and accurately make a button press with their right hand for “yes” to related pairs and “no” to unrelated pairs (Chou et al., 2009a,b). The stimuli were presented in an event-related design fashion. In the present study, the Optseq was used for automatically randomizing the sequence order and varying the inter-stimulus-interval (ISI) for rapid-presentation event-related fMRI experiments. The Optseq allowed more stimuli to be included within a given scanning interval at the cost of assuming that the overlap in the hemodynamic responses would be linear (http://surfer.nmr.mgh.harvard.edu/optseq, written by D. Greve, Charlestown, MA). In addition, previous fMRI studies using the OptSeq have got fairly stable findings across studies (Burock et al., 1998; Chou et al., 2006; Chou et al., 2009a,b; Bitan et al., 2009; Fan et al., 2010; Cao et al., 2011; Walter and Dassonville, 2011; Fan and Chou, 2012; Devereux et al., 2013; Mei et al., 2013; Saggar et al., 2014).

There were two kinds of control tasks. The perceptual control had 24 pairs of non-characters. Non-characters were created by replacing radicals of real characters with other radicals that did not form real Chinese characters such as “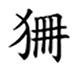,” “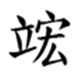,” and “
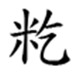
” (Wu and Chen, 2000). For the perceptual controls, trials consisted of a solid square (500 ms), followed by the first non-character (800 ms), a 200 ms blank interval, and the second non-character (3,000 ms). Participants determine whether the pair of stimuli were identical or not by pressing a yes or no button (Chou et al., 2009a,b). In the perceptual task, the non-characters were presented in different font sizes to encourage participants to perform the task based on the recognition of low-level visual similarity (Chou et al., 2009b). The second control task involved 24 baseline events. The participant was instructed to press a button when a solid square (1,300 ms) at the center of the visual field turned to a hollow square (3,000 ms) after a blank interval (200 ms; Chou et al., 2009a,b). The total number of trials was 120, and all conditions had the same 4,500 ms duration.

#### fMRI Data Acquisition

All participants received two practice sessions (one outside the scanner and the other one in the scanner) to ensure that they understood the task. The stimuli used for the practice sessions and the fMRI task were different. Each participant had to reach at least 80% accuracy for each practice session before entering the MRI task. In addition to the practice sessions, the children received a two-phase training session outside the scanner. In the first phase, the children and their parents were provided a brief introduction to each step of the scanning procedures. This phase was designed to help them understand the research aims to undergo the MRI investigation and the requirement to minimize head motion. They also received the samples of MRI sounds to experience the MRI task beforehand. In the second phase, the children were given a brief tour to the MRI environment, including looking at the magnet, MRI head coil, scanner bed, video goggles, and response box. After the tour, they entered the MRI scanning session. Earplugs were given to them to reduce noise. They lay in the scanner with a vacuum pillow to restrict head movement. Participants viewed visual stimuli projected onto a screen via a mirror attached to the head coil. Each participant performed two functional runs (Chou et al., 2009a,b). Images were acquired using a 3 Tesla Siemens Tim-Trio scanner with a 32-channel head coil. Each run took 4.7 minutes. Functional images were interleaved in an ascending order and were collected parallel to the anterior commissure-posterior commissure plane. The scanning parameters were the following: repetition time (TR) = 2000 ms; echo time (TE) = 24 ms; flip angle = 90°; matrix size = 64 × 64; field of view = 25.6 cm slice thickness = 3 mm; number of slices = 34. A high-resolution, T1-weighted three dimensional image was also acquired (Magnetization Prepared Rapid Gradient Echo, MP-RAGE; TR = 2300 ms; TE = 2.98 ms; flip angle = 9°; matrix size = 256 × 256; field of view = 25.6 cm; slice thickness = 1 mm). The orientation of the 3D image was identical to the functional slices. The task administration sequence was counterbalanced.

#### Conventional fMRI Analysis

Data analysis was performed using SPM8 (Statistical Parametric Mapping) with MATLAB version (Penny et al., 2007). The functional images were corrected for differences in slice-acquisition time to the middle slice of volume and were realigned to the first volume in the scanning session using affine transformations. No participant had more than 3 mm of movement in any plane. Co-registered images were normalized to the MNI (Montreal Neurological Institute) average template. Statistical analyses were calculated on the smoothed data (8 mm isotropic Gaussian kernel), with a high pass filter (128 s cut-off period) to remove low-frequency artifacts.

Data from each participant were entered into a general linear model using an event-related analysis procedure (Josephs and Henson, 1999). Character pairs were treated as individual events for analysis and modeled using a canonical Hemodynamic Response Function (HRF), and the stimulus onsets of the four experimental conditions (as a stick function) were convolved with the canonical HRF. There were four event types: related, unrelated, perceptual, and baseline. Thus, there were four regressors for these four experimental conditions in the General Linear Models (GLM). Parameter estimates from contrasts of the canonical HRF in single-subject models were entered into a random-effects analysis using one-sample *t*-tests across all participants to determine whether activation during a contrast was significant (i.e., parameter estimates were reliably greater than 0). Within each group, we used the contrast of the word (related and unrelated conditions) vs. baseline conditions in a whole-brain analysis (Liu et al., 2010). Based on common activation areas of within-group analyses for both groups, we conducted between-group comparisons. Also, for the contrasts between groups, we used the WFU PickAtlas^1^ to define ROI masks by selecting the anatomical masks of the left ventral IFG and left MTG based on our *a priori* hypothesis. The masks were used to control for multiple comparisons at *p* < 0.05 family wise error (FWE) corrected at the voxel level. For both the within-group and between-group analyses, all reported areas of activation were significant using *p* < 0.05 FWE corrected at the voxel level with a cluster size greater than 10 voxels. To examine developmental effects that were not due to differences in behavioral performance, we extracted the beta values from the peak voxels of brain regions from the between-group comparison (i.e., the left ventral IFG and left MTG). Then, we partialled out the effect of accuracy in the scanner as a covariate, and ANCOVAs were used to examine the differences in functional activation between groups. All reported results were significant using *p* < 0.025 corrected for two brain regions due to our *a priori* hypothesis.

#### Effective Connectivity Analysis

Three regions of interest (ROIs) were chosen from the present study using a more stringent threshold of *p* < 0.00005 uncorrected and containing a cluster size greater than or equal to 10 voxels. The threshold was suggested by a previous DCM article to enable the distinction among brain structures that otherwise comprise a single cluster (Bitan et al., 2007; Fan and Chou, 2012). The ROIs were the left ventral IFG (ventral IFG, BA 47), left posterior (MTG, BA 21), and left (FG, BA 37). All ROIs were 6-mm radius spheres centered on the most significant voxel in the individual’s activation map within 10-mm from the group maximum.

Effective connectivity analysis was used to examine connections among brain regions, and we used the Dynamic Causal Modelling (DCM) tool in SPM8 (Friston et al., 2003; Penny et al., 2004, 2007; Stephan et al., 2010). Our analysis adopted a two-stage procedure (Bitan et al., 2006; Cao et al., 2008). First, subject-specific DCMs were fully and reciprocally connected, with modulatory (bilinear) effects of the related and the unrelated conditions specified on coupling among all regions. In the DCM models, direct input was specified on FG in the visual modality; intrinsic connections were fully and reciprocally connected between the three ROIs (FG, MTG and IFG). The intrinsic connections between regions showed the interregional influences in the absence of modulating experimental effects (Friston et al., 2003), for example, lack of modulation on meaning judgments in this experiment. We restricted the results of intrinsic connections significant at a level of *p* < 0.008 (*p* < 0.05 corrected for six comparisons), tested in a 1-sample *t*-test (Bitan et al., 2005). However, we focused on the modulatory effects, which would be proper to reflect the experimental task (Friston et al., 2003), for example, the interregional influences on meaning judgments. In the second-stage, developmental differences between two groups in the modulatory effects for relatedness conditions were evaluated through three-way analysis of co variance (ANCOVAs): two group (adults, children) by two relatedness condition (related, unrelated) by two coupled connection (top-down, bottom-up) ANCOVAs, with the group as a between-factor, condition/connection as within-factors, and accuracy as a between-subject covariate. The bottom-up connections from the left FG and the top-down connections from the left IFG/MTG were examined due to previous adult studies using the same semantic task (Fan et al., 2010; Fan and Chou, 2012). This two-stage approach was the same as previous DCM studies of effective connectivity (Bitan et al., 2006; Cao et al., 2008; Kiran et al., 2015; Lahr et al., 2018).

#### Diffusion MRI Acquisition and Analysis

DSI was performed using a pulsed-gradient spin-echo diffusion echo-planar imaging sequence with a twice-refocused balanced echo (Reese et al., 2003). The scanning parameters were the following: TR = 9,600 ms; TE = 130 ms; bmax = 4,000 s/mm^2^; slice thickness = 2.5 mm; in-plane spatial resolution = 2.5 mm. To reduce the scan time, 102 diffusion-encoding directions were sampled on the grid points in the half-sphere of the q-space within a radius of 3.6 units (Wedeen et al., 2005). The acquired half-sphere data were projected to fill the other half of the sphere based on the fact that the data in the q-space were real and symmetrical about the origin.

**Figure 1:**
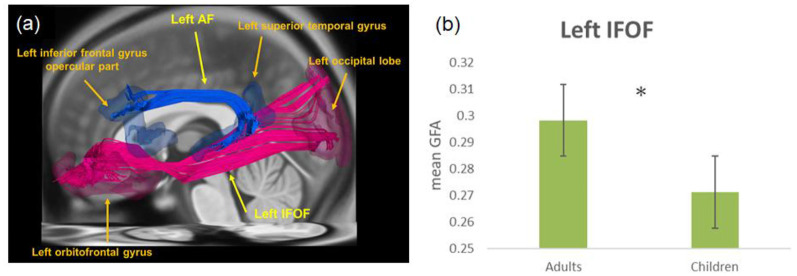
**(A)** The left inferior frontal-occipital fasciculus (IFOF), the arcuate fasciculus (AF), and their corresponding terminal regions in tractography, i.e., the left orbitofrontal gyrus and left occipital lobe for IFOF, as well as the left inferior frontal gyrus (IFG) opercular part and the left superior temporal gyrus (STG) for AF. The left ventral pathway connects the ventral IFG, middle temporal gyrus (MTG), and fusiform gyrus (FG; not shown) *via* IFOF. **(B)** The mean generalized fractional anisotropy (GFA) of IFOF in adults was significantly higher than that in children. *p < 0.05.

DSI tractography was performed based on a template-based approach to reconstruct fiber pathways of targeted tracts as described previously (Chen et al., 2015). In brief, Large Deformation Diffeomorphic Metric Mapping (LDDMM) and SPM8 were used to develop a group-specific DSI template from all individuals’ DSI datasets (Hsu et al., 2012). Second, we normalized the group-specific DSI template to a DSI template in MNI space, which was developed from 122 healthy participants’ DSI datasets and coregistered to MNI space (Hsu et al., 2015). The targeted tracts, i.e., the AF and the IFOF, were reconstructed on the DSI template using DSI Studio^2^ (Figure 1A). The reconstructed tracts were transformed from the DSI template in MNI space to an individual’s native DSI data through a group-specific DSI template by using LDDMM, and generalized fractional anisotropy (GFA) values were sampled along each of the targeted tracts. Similar to FA, GFA was used to infer the microstructural integrity of white matter fiber tracts (Gorczewski et al., 2009; Fritzsche et al., 2010). In the present study, we selected the GFA values for the AF and IFOF separately and calculated the mean GFA values of each pathway. Also, we treated the effect of accuracy in the scanner as a covariate, and ANCOVAs were used to examine the differences in structural connectivity between groups.

#### Statistical Analyses

We used IBM SPSS version 20.0 (SPSS Inc., Chicago, IL, USA) to conduct statistical analysis. The descriptive results were displayed as mean and SD for continuous variables. We conducted a two group (adults, children) by two relatedness condition (related, unrelated) ANOVA to examine the developmental differences between children and adults in behavioral performance. Moreover, ANCOVAs with partialing for accuracy were performed to examine the developmental differences between children and adults in functional activation, structural connectivity, and effective connectivity. Moreover, multiple regressions were used to examine the relationship between age in months and behavioral performance, structural connectivity, and effective connectivity, including accuracy as a between-subject covariate. This approach allowed us to examine age-related increases or decreases in both activation and connectivity that were independent of accuracy differences.

## Results

### Behavioral Results

In the adult group, accuracy (mean ± SD) for the related and unrelated conditions was 95 ± 5% and 99 ± 2%, respectively. In the child group, accuracy (mean ± SD) for the related and unrelated conditions was 71 ± 19% and 94 ± 9%, respectively. An ANOVA with two groups (adults, children) by two relatedness condition (related, unrelated), with the group as a between-factor and with the condition as within-factors, showed that the main effect of group was significant (*F*_(1,50)_ = 40.30, *p* < 0.01). The main effect of condition was significant (*F*_(1,50)_ = 45.15, *p* < 0.01). The interaction of group and condition was significant (*F*_(1,50)_ = 25.51, *p* < 0.01). To further understand the two-way interaction, *t*-tests showed that adults were more accurate than children in both the related and unrelated conditions (*t*_(50)_ = 6.28, *p* < 0.01; *t*_(50)_ = 2.64, *p* < 0.01, respectively).

In the adult group, reaction times (mean ± SD) for the related and unrelated conditions were 824 ± 154 ms and 881 ± 143 ms, respectively. In the child group, reaction times (mean ± SD) for the related and unrelated conditions were 1,284 ± 235 ms and 1,188 ± 226 ms, respectively. A two group (adults, children) by two relatedness condition (related, unrelated) ANOVA showed that the main effect of group was significant (*F*_(1,50)_ = 58.08, *p* < 0.01). The main effect of condition was not significant (*F*_(1,50)_ = 1.05 *p* = 0.31). The interaction of group and condition was significant (*F*_(1,50)_ = 16.24, *p* < 0.01). To further understand the two-way interaction, *t*-tests showed that adults were significantly faster than children in the related and unrelated conditions (*t*_(50)_ = −8.35, *p* < 0.01; *t*_(50)_ = −5.85, *p* < 0.01, respectively).

Accuracy (mean ± SD) for the baseline conditions in the adults and children was 99 ± 2% and 98 ± 5%, respectively. Reaction times (mean ± SD) for the baseline conditions in the adults and children were 584 ± 143 ms and 786 ± 141 ms, respectively.

### Conventional fMRI Results

**Figure 2:**
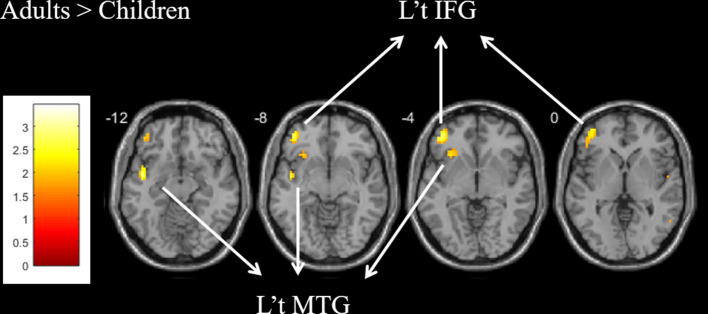
For the contrast of the word vs. baseline condition, greater activation was found in the left ventral IFG and MTG for the adult group as compared with the child group.

For the contrast of “word vs. baseline condition” within the group, both groups showed common activation in the left ventral IFG (BA 47) and left MTG (BA 21). The direct comparison between groups showed that the adults produced greater activation than children in the left ventral IFG (BA 47; voxels = 54; *x* = −42, *y* = 44, *z* = −5; *Z* = 2.72) and MTG (BA 21; voxels = 38; *x* = −57, *y* = −55, *z* = −5; *Z* = 2.42; Figure 2 and Supplementary Table S1). The child group showed less activation than the adult group. There was no group difference in the left dorsal IFG. There was no significant correlation between brain activation and age within children.

In order to examine age-related increases or decreases in activation, we extracted beta-values from the left ventral IFG and MTG in the contrast of “word vs. baseline condition,” and ANCOVAs were performed with partialing out accuracy as a covariate (*p* < 0.025 equal to *p* < 0.05 corrected for two comparisons). After partialing out the effect of accuracy, significantly greater beta-values were found in the left ventral IFG (*F*_(1,49)_ = 6.10, *p* = 0.017) and MTG (*F*_(1,49)_ = 5.75, *p* = 0.020) for adults relative to children.

### Structural Connectivity Differences Between the Adult and Child Groups

With partialing out the effect of accuracy in the scanner as a covariate, group comparisons revealed a significant difference in the left inferior frontal occipital fasciculus (*F*_(1,49)_ = 23.36, *p* = 0.000), showing greater mean GFA for adults as compared to children (Figure 1B). For the left AF, there was no statistically significant group difference (*F*_(1,49)_ = 0.55, *p* = 0.460).

Within children, there was a positive correlation between mean GFA of the left inferior frontal occipital fasciculus and age (*r* = 0.574, *p* = 0.003) with partialing out the effect of accuracy in the scanner as a covariate. Conversely, the mean GFA of the left AF was not significantly correlated with age within children (*r* = 0.061, *p* = 0.774).

### Effective Connectivity Differences Between the Adult and Child Groups

**Figure 3:**
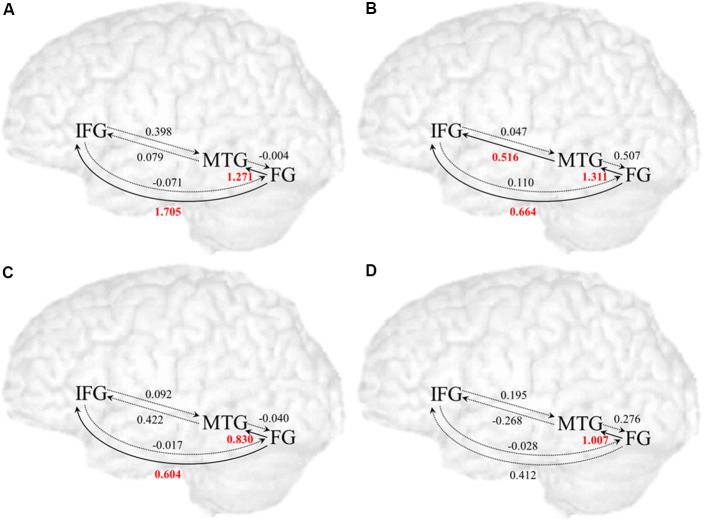
Modulatory effects in the related and unrelated conditions among all regions for the adult and child group. **(A)** Modulatory effects in the related condition for the adult group. **(B)** Modulatory effects in the unrelated condition for the adult group. **(C)** Modulatory effects in the related condition for the child group. **(D)** Modulatory effects in the unrelated condition for the child group. Solid line arrows indicate significant influences on modulated connections (*p* < 0.008 with *p* < 0.05 corrected for six comparisons), and dotted line arrows indicate non-significant connections.

Table 1 and Figure 3 presents the posterior means of modulatory effects of the related and unrelated conditions for both the adult and the child groups. One-sample *t*-tests were used to examine whether each modulatory effect was significantly different from zero (*p* < 0.008 equal to *p* < 0.05 corrected for six comparisons). Also, all intrinsic connections were significant (Supplementary Table S2).

**Table 1:** The posterior means of the parameter densities on modulatory effects for the adult and child groups for the related and unrelated conditions among all regions (IFG, ventral inferior frontal gyrus; MTG, posterior middle temporal gyrus; FG, fusiform gyrus).

Adults related condition				Adults unrelated condition			
*From*:	IFG	MTG	FG	*From*:	IFG	MTG	FG
*To*:				*To*:			
IFG		0.079	1.705*	IFG		0.516*	0.664*
MTG	0.398		1.271*	MTG	0.047		1.311*
FG	−0.071	−0.004		FG	0.110	0.507	
**Children related condition**				**Children unrelated condition**			
*From*:	IFG	MTG	FG	*From*:	IFG	MTG	FG
*To:*				*To*:			
IFG		0.422	0.604*	IFG		−0.268	0.412
MTG	0.092		0.830*	MTG	0.195		1.007*
FG	−0.017	−0.040		FG	−0.028	0.276	

Note: *p < 0.008 (*p* < 0.05 corrected for six comparisons).

**Figure 4:**
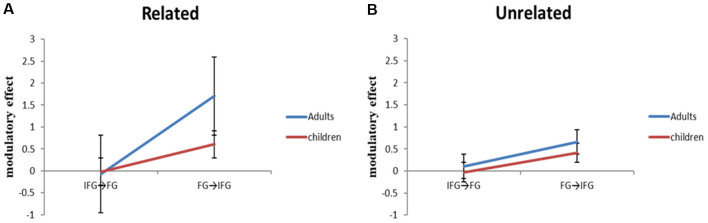
The adult group showed significantly stronger modulatory effects than the child group on the bottom-up connection from the fusiform gyrus (FG) to ventral inferior frontal gyrus (IFG), but not on the top-down connection from the IFG to FG. This difference was found in the related condition, but not in the unrelated condition.

With accuracy as a covariate, we calculated a two group (adults, children) by two relatedness condition (related, unrelated) by two coupled connection (top-down from the ventral IFG to the FG, bottom-up from the FG to the ventral IFG) ANCOVA to investigate the interaction between the top-down region (ventral IFG) and bottom-up region (FG; Figure 4). The three-way interaction of group (adults, children) × relatedness condition (related, unrelated) × coupled connection (top-down from the ventral IFG to the FG, bottom-up from the FG to the ventral IFG) was significant (*F*_(1,49)_ = 5.57, *p* = 0.022). To further understand the three-way interaction, a two group (adults, children) by two coupled connection (ventral IFG to FG, FG to ventral IFG) ANCOVA was calculated for related and unrelated conditions separately. The analyses revealed that there were significant interactions of group × direction (*F*_(1,49)_ = 5.99, *p* = 0.018), and main effect of group (*F*_(1,49)_ = 5.23, *p* = 0.027) in the related condition, but not in the unrelated condition. To further understand the two-way interaction in the related condition, *t*-tests revealed that the adults showed a significantly larger modulatory effect in the bottom-up connection from the FG to the ventral IFG than in the top-down connection from the ventral IFG to the FG (*t*_(26)_ = −6.59, *p* = 0.000) and that the bottom-up effect from the FG to the ventral IFG was stronger in adults as compared to children (*t*_(50)_ = 3.74, *p* = 0.000). Moreover, with partialing out the effect of accuracy as a covariate, there was a positive correlation between the modulatory effect from the FG to the ventral IFG and age within children in the related condition (*r* = 0.431, *p* = 0.031).

With accuracy as a covariate, we also calculated a two group (adults, children) by two relatedness condition (related, unrelated) by two coupled connection (top-down from MTG to FG, bottom-up from FG to MTG) ANCOVA to investigate the interaction between the top-down region (MTG) and bottom-up region (FG). The analysis only revealed a marginal main effect of direction (*F*_(1,49)_ = 3.75, *p* = 0.059) with the bottom-up effect from the FG to MTG being stronger than the top-down effect from the MTG to FG.

### Correlations Between Structural Connectivity and Brain Activation/Effective Connectivity

For the correlation between GFA and activation, partialing out the effect of accuracy as a covariate, there was a positive correlation between mean GFA of the left inferior frontal occipital fasciculus and the ventral IFG (*r* = 0.337, *p* = 0.016). For the correlation between GFA and connection strength, partialing out the effect of accuracy as a covariate, stronger mean GFA of the left inferior frontal occipital fasciculus was positively correlated with the modulatory effect from the FG to the ventral IFG in the related condition (*r* = 0.313, *p* = 0.025), and with the modulatory effect from the FG to the MTG in the related condition (*r* = 0.297, *p* = 0.036).

## Discussion

To the best of our knowledge, the present study is the first to investigate developmental differences in semantic processing regarding brain activation, structural connectivity, and effective connectivity between adults and children. Compared to children, adults showed significantly better behavioral performance, greater functional activation in the left ventral IFG and MTG, and greater mean GFA in the left ventral pathway (IFOF). Moreover, the bottom-up connection from the left FG to the ventral IFG was significantly stronger in adults than that in children for the related condition, but not for the unrelated condition. These findings provide converging evidence to understand the developmental differences between adults and children during semantic processing in Chinese.

In the present study, adults exhibited significantly greater activation in the left ventral IFG (BA 47) and MTG (BA 21) than children, implying maturation in semantic processing *via* the retrieval or selection of appropriate semantic knowledge (Poldrack et al., 1999; Wagner et al., 2001). Previous studies have suggested that the ventral IFG is associated with access, retrieval, selection, and gating of semantic information, while the MTG may play a role in storing conceptual features that are associated with lexical representations (Hickok and Poeppel, 2007; Martin, 2007; Sonty et al., 2007). In the present study, however, adults did not show increased activation in the dorsal IFG as compared to children. This finding implies that the dorsal IFG may not play a significant role in the developmental differences of semantic processing.

In the present study, a greater mean GFA of the left ventral pathway (IFOF) was found in adults; however, we did not find a group difference in the left dorsal pathway (AF). The findings suggest maturation in the ventral pathway. Previous studies have found that the ventral pathway is involved in lexical-semantic processing (Glasser and Rilling, 2008; Saur et al., 2008), and the dorsal pathway (AF) is involved in phonological processing (Saur et al., 2008). For language functions, both pathways are not mutually exclusive but rather work in parallel (Rauschecker and Scott, 2009). Also, previous DTI studies have proposed that age-related increases in FA may be involved in axonal myelination for both the dorsal and ventral pathways (Perani et al., 2011). However, we only found a positive correlation between the mean GFA of the left ventral pathway (IFOF) and age within children, suggesting an age-related increase in microstructural integrity of the ventral pathways. As their age increased, elder children may have greater microstructural integrity in the ventral pathways as compared to younger children.

From effective connectivity results, we found that the bottom-up connection from the left FG to ventral IFG was significantly stronger in adults than that in children for the related condition, but not for the unrelated condition. Previous studies have suggested that the ventral IFG is associated with retrieval or selection of appropriate semantic knowledge (Thompson-Schill et al., 1999; Wagner et al., 2001), while the FG is involved in visual-orthographic processing (Nakamura et al., 2000; Lin et al., 2007). Orthographic processing is generally attributed to the FG, which has been reliably shown to be responsible for visually presented words (McCandliss et al., 2003). Moreover, older children may more rely on orthographic pattern separation showing greater activation in the FG than younger children (McNorgan et al., 2011). Taken together, the left ventral IFG is implicated in controlling semantic retrieval, while the FG is implicated in processing orthographic representations (Lau et al., 2008). Therefore, our finding of a stronger FG-ventral IFG connection in adults suggests that orthographic processing in the FG may have a strong link with semantic retrieval in the ventral IFG in adults than in children. More importantly, we found a positive correlation between the FG-ventral IFG connection and age within children. This correlation provides further evidence of the mapping from orthography to semantics with age during semantic processing in Chinese.

The findings and interpretations of the present study should be considered in light of its limitation. Although there were no significant differences of head motion in translation and rotation between children and adults (x-translation: *t*_(50)_ = 0.63, *p* > 0.05; y-translation: *t*_(50)_ = −1.18, *p* > 0.05; z-translation: *t*_(50)_ = −1.15, *p* > 0.05; pitch: *t*_(50)_ = 1.37, *p* > 0.05; roll: *t*_(50)_ = 1.94, *p* > 0.05; yaw: *t*_(50)_ = −0.03, *p* > 0.05, respectively), the age-related head size might be a potential confound. Therefore, a larger sample with a wider age range from childhood to adulthood is needed to further evaluate the structure and function of the neural mechanism underlying semantic processing across different developmental stages. Moreover, in addition to the cross-sectional studies, a prospective longitudinal study investigating the trajectory of developmental changes in semantic processing is warranted.

In conclusion, combining fMRI, DSI, and DCM provides a complete insight into the developmental differences between adults and children during semantic processing in Chinese. Our findings suggest that age-related maturation in brain activation (ventral IFG and MTG), structural connectivity (IFOF), and effective connectivity (from FG to ventral IFG) might be associated with the bottom-up influences of orthographic representations on retrieving semantic representations for processing Chinese characters. Our results clearly contribute to our knowledge about developmental differences, which are not only associated with structural maturation but also with functional brain activation. Most importantly, our findings of effective connectivity revealed that adults might be better able to utilize visual-orthographic word information of the fusiform cortex during Chinese meaning judgments. These findings might advance our knowledge of the communication between brain regions for language functions, especially for adults and children in Chinese.

## Data Availability Statement

The datasets generated for the present study are available on request to the corresponding author.

## Ethics Statement

The studies involving human participants were reviewed and approved by National Taiwan University Hospital Research Ethics Committee. Written informed consent to participate in this study was provided by the participants’ legal guardian/next of kin.

## Author Contributions

L-YF, W-YT, and T-LC designed research. L-YF, Y-CL, Y-CH, and Y-JC decided upon and performed the analyses. L-YF drafted the main manuscript text. All authors reviewed the manuscript and approved it for submission.

## Conflict of Interest

The authors report no biomedical financial interests or potential conflicts of interest.

The handling Editor declared a past co-authorship with one of the authors W-YT.

## Footnotes

^1^https://www.nitrc.org/projects/wfu_pickatlas/

^2^http://dsi-studio.labsolver.org
