# Supplementary material for: Developmental Differences of Structural Connectivity and Effective Connectivity in Semantic Judgments of Chinese Characters
Source: Front Hum Neurosci. 2020 Jun 30;14:233. doi: 10.3389/fnhum.2020.00233 (PMC7344167; doi:10.3389/fnhum.2020.00233)
Supplement: Supplementary file 1 [file Data_Sheet_1.PDF]

Supplementary Table 1. Brain activation within/between adult and child groups.

Cortical regions	H	BA	Voxels	Z test	MNI coordinates		
					x	y	z
Adults							
Inferior frontal gyrus	L	45/47	600	7.20	-48	38	1
Superior frontal gyrus	L	6	76	6.30	-3	8	58
Middle temporal gyrus	L	21	123	3.17	-54	-46	4
Fusiform gyrus	L	37	59	4.73	-39	-52	-20
Children							
Inferior frontal gyrus	L	47	196	4.28	-54	20	-11
Superior frontal gyrus	L	6	190	4.85	-3	8	58
Middle temporal gyrus	L	21/22	99	3.17	-54	-40	1
Fusiform gyrus	L	37	107	5.43	-42	-73	-17
Adults > Children							
Inferior frontal gyrus	L	47	54	2.72	-42	44	-5
	L	45	19	2.97	-48	32	16
Middle temporal gyrus	L	21	11	2.88	-45	-1	-11
	L	21	38	2.42	-57	-55	-5
Children > Adults							

None

Note: H, hemisphere; L, left; R, right; BA, Brodmann's area; Voxels, number of voxels in cluster at $p < .05$ for FWE (familywise error) corrected; Coordinates of activation peak(s) within a region based on a z test are given in the MNI stereotactic space (x, y, z).

Supplementary Table 2. All intrinsic connections for the adult and child groups among all regions (IFG: ventral inferior frontal gyrus, MTG: posterior middle temporal gyrus, FG: fusiform gyrus).

**Adults**			
From:	IFG	MTG	FG
To: IFG		**0.199***	**0.254***
MTG	**0.197***		**0.349***
FG	**0.182***	**0.116***	
**Children**			
From:	IFG	MTG	FG
To: IFG		**0.199***	**0.140***
MTG	**0.206***		**0.324***
FG	**0.163***	**0.175***	

Note: *$p < .008$ ($p < .05$ corrected for 6 comparisons)
